# Supplementary material for: Relationship between tobacco use and body mass index- a propensity score matching analysis of an indian National Survey
Source: PLoS One. 2025 May 28;20(5):e0323274. doi: 10.1371/journal.pone.0323274 (PMC12118988; doi:10.1371/journal.pone.0323274)
Supplement: S1 Table — (DOCX) [file pone.0323274.s001.docx]

**S1 Table: Multinomial binary logistic regression analysis depicting the likelihood of being underweight or overweight/obese in participants with or without tobacco use (any form).**

| **Covariates** | **Underweight** | | **Overweight** | |
| --- | --- | --- | --- | --- |
|  | **Adjusted Odds ratio**  **(95% CI)** | **p-value** | **Adjusted Odds ratio**  **(95% CI)** | **p-value** |
| **Tobacco usage** |  |  |  |  |
| No | Ref value |  | Ref value |  |
| yes | 1.2 (1.2-1.2) | <0.001 | 0.9 (0.9-0.9) | <0.001 |
| **Age (Completed years)** |  |  |  |  |
| 15-24 | Ref value |  | Ref value |  |
| 25-34 | 0.6 (0.6-0.7) | <0.001 | 2.1 (2.1-2.2) | <0.001 |
| 35-44 | 0.5 (0.5-0.5) | <0.001 | 3.3 (3.2-3.3) | <0.001 |
| 45-54 | 0.5 (0.5-0.5) | <0.001 | 3.6 (3.6-3.7) | <0.001 |
| **Sex** |  |  |  |  |
| Male | Ref value |  | Ref value |  |
| Female | 1.3 (1.3-1.4) | <0.001 | 0.9 (0.9-0.9) | <0.001 |
| **Place of Residence** |  |  |  |  |
| Urban | Ref value |  | Ref value |  |
| Rural | 1.1 (1-1.1) | <0.001 | 0.9 (0.9-0.9) | <0.001 |
| **Region of India** |  |  |  |  |
| North | Ref value |  | Ref value |  |
| Central | 1.1 (1.1-1.1) | <0.001 | 0.9 (0.9-0.9) | <0.001 |
| East | 1.2 (1.2-1.3) | <0.001 | 0.9 (0.9-1) | <0.001 |
| Northeast | 0.7 (0.7-0.7) | <0.001 | 1 (1-1.1) | 0.001 |
| West | 1.8 (1.7-1.8) | <0.001 | 0.8 (0.8-0.8) | <0.001 |
| South | 1.4 (1.4-1.4) | <0.001 | 1.3 (1.3-1.4) | <0.001 |
| **Highest Education** |  |  |  |  |
| No Education | Ref value |  | Ref value |  |
| Primary | 0.9 (0.9-0.9) | <0.001 | 1.1 (1.1-1.2) | <0.001 |
| Secondary | 0.9 (0.9-0.9) | <0.001 | 1.3 (1.3-1.3) | <0.001 |
| Higher | 0.6 (0.6-0.6) | <0.001 | 1.4 (1.4-1.4) | <0.001 |
| **Marital status** |  |  |  |  |
| Never Married | Ref value |  | Ref value |  |
| Currently Married | 0.6 (0.6-0.6) | <0.001 | 1.7 (1.7-1.8) | <0.001 |
| Widowed/Divorced/Separated | 0.7 (0.6-0.7) | <0.001 | 1.5 (1.5-1.6) | <0.001 |
| **Religion** |  |  |  |  |
| Hindu | Ref value |  | Ref value |  |
| Muslim | 0.8 (0.7-0.8) | <0.001 | 1.2 (1.2-1.2) | <0.001 |
| Other | 0.7 (0.7-0.7) | <0.001 | 1.3 (1.3-1.3) | <0.001 |
| **Social Caste** |  |  |  |  |
| Scheduled Caste | Ref value |  | Ref value |  |
| Scheduled Tribe | 0.9 (0.9-0.9) | <0.001 | 0.8 (0.8-0.9) | <0.001 |
| Other Backward Caste | 1 (1-1) | 0.14 | 1 (1-1) | <0.001 |
| Others | 0.9 (0.9-0.9) | <0.001 | 1.1 (1.1-1.2) | <0.001 |
| **Wealth Quintile** |  |  |  |  |
| Poorest | Ref value |  | Ref value |  |
| Poorer | 0.8 (0.8-0.9) | <0.001 | 1.4 (1.4-1.5) | <0.001 |
| Middle | 0.7 (0.7-0.8) | <0.001 | 1.9 (1.9-1.9) | <0.001 |
| Richer | 0.7 (0.7-0.7) | <0.001 | 2.4 (2.3-2.4) | <0.001 |
| Richest | 0.6 (0.6-0.6) | <0.001 | 3 (3-3.1) | <0.001 |
| Constant | 0.7 (0.7-0.8) | <0.001 | 0.1 (0.1-0.1) | <0.001 |
